# Magnetically induced enzymatic cascades – advancing towards multi-fuel direct/mediated bioelectrocatalysis†

**DOI:** 10.1039/c8na00346g

**Published:** 2019-02-28

**Authors:** Katharina Herkendell, Andreas Stemmer, Ran Tel-Vered

**Affiliations:** ETH Zürich, Nanotechnology Group Säumerstrasse 4, CH-8803 Rüschlikon Switzerland teran@ethz.ch

## Abstract

A generic method to magnetically assemble enzymatic cascades on electrode surfaces is introduced. The versatile method enables the simultaneous activation of both direct and mediated electron transfer bioelectrocatalysis to harness different substrates, which can serve as multiple fuels and oxidizers in biofuel cells generating clean energy.

## Abstract 2

Enzyme-functionalized magnetic nanoparticles are channeled by magnetic field gradients onto modified electrodes to activate enzymatic cascades for multi-substrate power generation.

## Graphical abstract

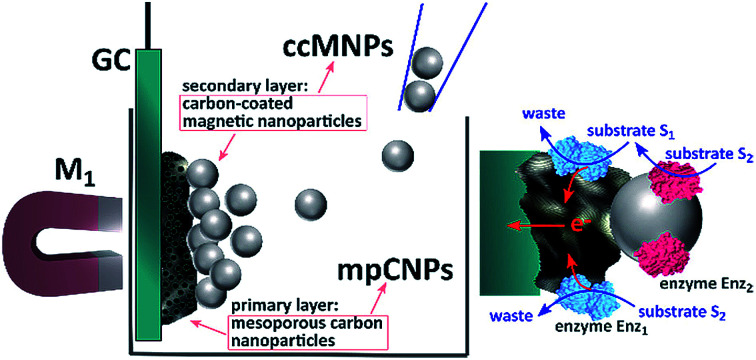

Despite the ongoing progress in the fields of bioelectrochemistry and materials science over the last few decades, several practical issues hinder the implementation of nano-engineered enzymatic catalytic electrode assemblies in targeted environments. The operation, for example, of biofuel cells under rapidly changing conditions in low accessibility destinations and in highly heterogeneous fluids is of primary importance for application in medical or environmental fields.

Here, we present a versatile method to increase the extractable power from a heterogeneous fuel solution by inducing enzymatic cascades with external magnetic fields. The use of magnetic fields is known to activate^1–5^ and even to enhance bioelectrocatalytic systems.^6–11^ Recently, modified magnetic nanoparticles, MNPs, are increasingly applied as biocatalyst carriers, as their ability to be infused into, dispersed in, or collected from solutions by an external magnetic field enables control over enzymatic catalysis in otherwise inaccessible environments. Magnetically induced shuttling of enzymes immobilized on MNPs was implemented, for example, to perform selective biocatalysis,^12,13^ to construct enzymatic logic circuits,^14^ and to enhance the operational lifetime of enzymatic biofuel cells.^15^ In this regard, carbon or gold coatings, and modifications with alkyl chains were demonstrated in the past to enhance the immobilization of enzymes on MNPs.^16–18^ In the current work, we employ carbon coated magnetic nanoparticles, ccMNPs, separately modified with different enzymes, to induce cascaded processes on the electrode surfaces. The magnetically induced enzymatic cascades facilitate a mass transfer communication between the adjacent enzymes yielding direct or mediated electrical wiring between the respective primary modified enzymatic layer and the underlying conductor surface. The biofunctionalized electrodes are used to construct biofuel cells operating in different environments and allowing the consumption of different fuel and oxidizer substrates in the production of electrical power. The generality and versatility of the method are demonstrated with different examples of anodic and cathodic bioelectrocatalysis, where direct or mediated charge transfer architectures are implemented.

Scheme 1 depicts the method employed to magnetically induce on-demand enzymatic cascaded transformations on glassy carbon, GC, surfaces.‡‡Scheme 1 contains enzymatic structures that were visualized with Jmol: an open-source Java viewer for chemical structures in 3D. http://www.jmol.org/ To this end, we use two populations of carbon nanoparticles. The first is composed of mesoporous carbon nanoparticles, mpCNPs, on which the redox enzyme, Enz_1_, is loaded and immobilized, see ESI† for further details. The mpCNPs act as a high-surface-area, electrically conductive matrix that assists the electronic conductance between the immobilized enzymatic cofactors and the underlying GC support. Depending on the enzymatic composition of the assembly, the electron transfer process may be either direct (DET) or mediated (MET), where in the latter case the hosting of relay molecules inside the pores and their capping by specific enzymes enable a pore-confined diffusion process that yields an effective bioelectrocatalysis.^19–22^ The second population of carbon nanoparticles, required for the magnetic activation of the cascaded biosystem, is composed of ccMNPs on which the cascadic counterpart of enzyme 1, namely Enz_2_, is immobilized. The introduction of the Enz_2_-modified ccMNPs into a buffer solution, where the Enz_1_-functionalized mpCNPs/GC assembly is immersed, followed by the activation of a magnetic field gradient attracting the magnetic nanoparticles onto the electrode surface, is shown to generate a path of electrical conductivity for the transfer of redox electrons associated with the energetic downhill enzymatic transformations in the assemblies. To exemplify the generic applicability of the method, we demonstrate a set of three cascade systems; two involve DET in anodic or cathodic directionalities, and one depicts a MET anodic system, as summarized in Table 1.

**Scheme 1:**
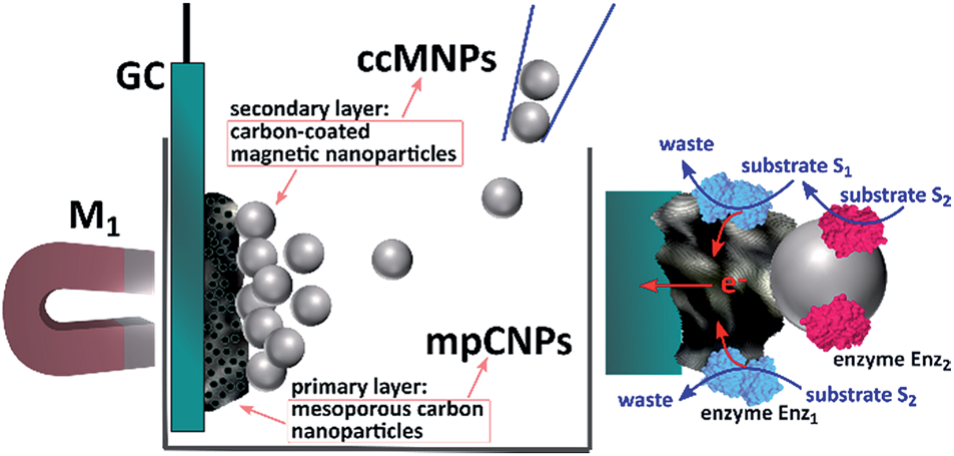
Illustration of the magnetic activation of bioelectrocatalytic cascades through channeling of enzyme-modified magnetic nanoparticles onto the surfaces of enzyme-functionalized mesoporous carbon nanoparticles.

**Table 1:** The systems used to demonstrate the induction of cascaded bioelectrocatalysis, consisting of a primary layer with enzyme 1 immobilized on a mpCNP matrix on top of a GC current collector, and a magnetically inducible secondary layer based on ccMNPs modified with enzyme 2

Assembly	Enzyme 1 (Enz_1_)	Substrate 1 (S_1_)	Mediator	*Γ*_enzyme 1_ pmol cm^−2^	Enzyme 2 (Enz_2_)	Substrate 2 (S_2_)	*Γ*_enzyme 2_ pmol mg_ccMNP_^−1^	*Γ*_enzyme 2_ pmol cm^−2^
I. Cathodic DET	BOD	O_2_	—	6.0 ± 1.0	CAT	H_2_O_2_	2.5 ± 0.1	4.0 ± 0.2
II. Anodic DET	FDH	Fructose	—	3.8 ± 0.9	INV	Sucrose	4.1 ± 0.6	6.3 ± 1.0
III. Anodic MET	HRP	H_2_O_2_	MB	9.2 ± 1.3	GOx	Glucose	2.2 ± 0.2	3.4 ± 0.4

Fig. 1(A) shows the cyclic voltammograms measured for assembly I in Table 1, showing a DET cathodic system based on a cascade between bilirubin oxidase (BOD) and catalase (CAT). Evidently, when testing the BOD-functionalized mpCNPs/GC assembly alone, in the absence of the CAT-modified ccMNPs and in the absence of O_2_, only a background charging current is observed, curve (Enz_1_S_0_). All amperometric responses are reported as current densities normalized to the geometrical surface area (*A* = 0.32 cm^2^) of the immersed GC current collector. At this stage, addition of H_2_O_2_, which is the substrate of Enz_2_ that is still absent in the assembly, results in a diminished current, curve (Enz_1_S_2_), implying that the contribution of this substance to the electrocatalysis in the absence of the second biocatalyst is negligible. Upon purging the N_2_-saturated electrolyte with O_2_, a relatively small bioelectrocatalytic cathodic current with an onset potential of *E* = 0.55 V *vs.* SCE is obtained, curve (Enz_1_S_1_). This current corresponds to the BOD-electrocatalyzed reduction of oxygen to water, in accordance with:

1 O_2_ + 4H^+^ + 4e^−^ → 2H_2_O (on Enz_1_ = BOD)

**Fig. 1:**
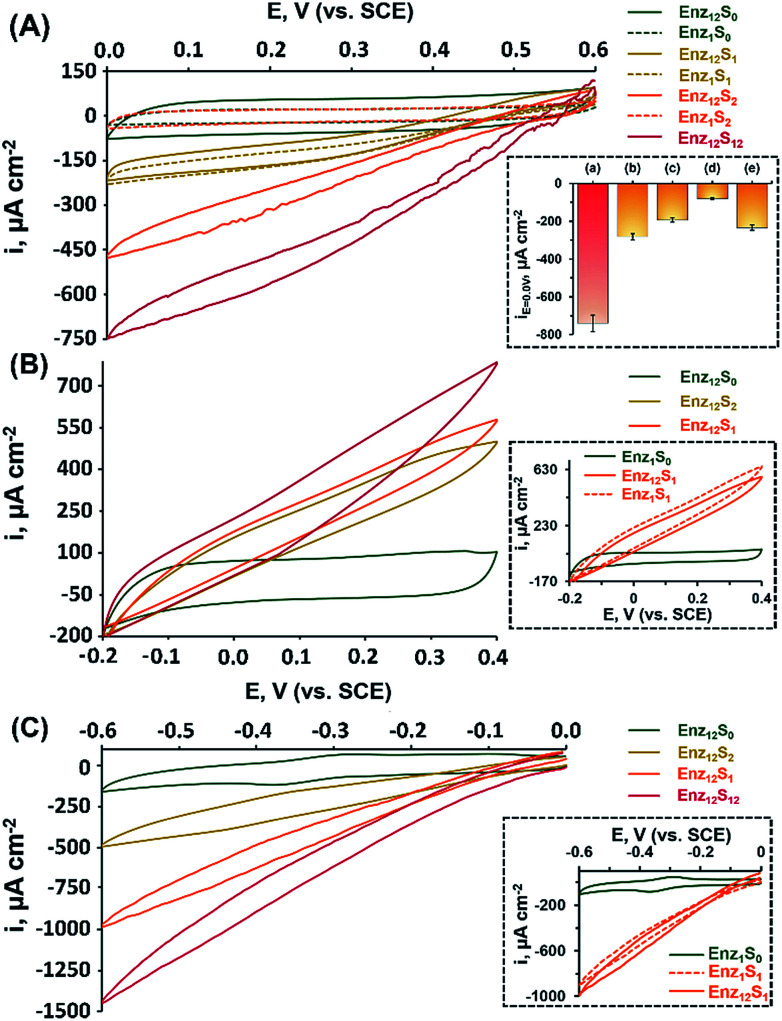
Bioelectrocatalytic currents observed *via* cyclic voltammetry: (A) assembly I (BOD-CAT), (B) assembly II (FDH-INV), and (C) assembly III (HRP(MB)-GOx). Curves (Enz_12_) show the systems' responses following the application of the magnetically activated secondary layer: (S_0_) in the absence of fuels; (S_1_) in the presence of substrate 1, *c*_O_2__ = saturated, *c*_fructose_ = 200 mM, or *c*_H_2_O_2__ = 60 mM; (S_2_) in the presence of substrate 2, *c*_H_2_O_2__ = 40 mM, *c*_sucrose_ = 200 mM, or *c*_glucose_ = 200 mM; and (S_12_) in the presence of both substrates 1 and 2. Curves (Enz_1_) show, for comparison, the systems' responses with solely the primary layer containing enzyme 1 applied. Control experiments in (A) inset: both layers with (a) a magnet applied, (b) no magnet applied, (c) reversed assembly (CAT on mpCNPs and BOD on ccMNPs), (d) like (a), but for a denatured assembly (after 30 min at 90 °C), and (e) a magnet applied on the opposite side of the cell. The error bars correspond to a set of *N* = 3 experiments. All measurements were performed at a scan rate of 10 mV s^−1^ in 100 mM MES buffer (pH 5.5).

Similar to other carbonaceous nanostructures,^23–26^ DET between the T1 blue copper center of BOD and the mpCNP matrix occurs,^27,28^ enabling electrocatalysis in the absence of any additional mediating relays. At this stage, CAT-loaded ccMNPs were introduced into the electrolyte solution concurrently with a magnet placed behind the BOD-functionalized mpCNPs/GC assembly. The attachment of the CAT-ccMNPs to the mpCNPs/GC facilitated by the magnetic field allowed the coupling of the cascade counterparts, which moderately increased the charging current in the absence of O_2_, curve (Enz_12_S_0_). As expected, in the presence of the O_2_ fuel alone, the bioelectrocatalytic response of the CAT-ccMNP-magnetized mpCNPs/GC assembly, curve (Enz_12_S_1_), was quite identical to that of the mpCNPs/GC assembly alone, indicating that the diffusion paths of O_2_ to BOD remain open upon the magnetic attachment of the ccMNPs to the electrode surface. Curve (Enz_12_S_2_) depicts the challenge of the magnetized assembly with the substrate of CAT, H_2_O_2_, 40 mM, alone. As this enzyme catalyzes the decomposition of hydrogen peroxide into water and oxygen, eqn (2), a local buildup of O_2_, above the limiting solubility of this species in the buffer solution,^19,21^ occurs in the vicinity of the BOD-modified surface. This is evident from the O_2_ bubble formation at the electrode surface and the subsequent fluctuation in electrocatalytic currents due to transient changes of the effective surface area exposed to the electrolyte.

2 2H_2_O_2_ → 2H_2_O + O_2_ (on Enz_2_ = CAT)

The bioelectrocatalytic current, *ca.* −470 μA cm^−2^, that follows the reduction of the substrate in this case is thus significantly more intense than in curve Enz_12_S_1_, whereas the noisier pattern of the scan reflects changes to the effective surface area of the electrode caused by the formation of O_2_ bubbles through the excess enzymatic product. Evidently, even a higher bioelectrocatalytic cathodic current, *ca.* −750 μA cm^−2^, is observed in the presence of both the O_2_ and H_2_O_2_ substrates in the magnetically induced assembly, curve Enz_12_S_12_. The bioelectrocatalytic response of the magnetically assembled cascade in the presence of the two substrates was then compared to those of different related assemblies, with the relative amperometric responses at *E* = 0.0 V *vs.* SCE depicted in Fig. 1(A), inset. Bar (b) of the inset reveals the bioelectrocatalytic current obtained on the BOD-mpCNPs/GC assembly in the presence of the CAT-loaded ccMNPs in the cell, yet without a magnetic field gradient applied. As can be seen, compared to the magnetically attached cascade, bar (a), a lower catalytic current of *ca.* −280 μA cm^−2^ is obtained, highlighting the importance of the proximity between the enzymes in the generation of effective cascading transformations. In another experiment, we reversed the construction of the assembly, and magnetically attached BOD-loaded ccMNPs onto a primary layer consisting of CAT-functionalized mpCNPs. As can be seen from the amperometric response, bar (c), a significantly lower current, as compared to bar (a), was evident. We attribute these findings to both the insulating layer of the redox inactive CAT, now covering the primary layer and hindering the seamless charge transfer to the base GC collector, and the reduced loading of the BOD bioelectrocatalysts on the ccMNPs as compared to the mpCNP layer, see Table 1 and the ESI (Table S1, Fig. S1(A)–(E)†), detailing the relative surface coverage values measured and their derivation. The next experiment involved the preliminary denaturation of the primary BOD-mpCNP layer by a heat treatment at 90 °C for 30 minutes. The result, shown in bar (d), indicates that only a diminished current could be obtained due to the temperature-induced inactivation of the electroactive biocatalyst in the system. The magnetically induced dissociation of the attached secondary CAT-loaded ccMNP layer and its effect on the bioelectrocatalytic responses were also tested, bar (e). Upon the translocation of the magnet exerting the associative field between the cascading elements to the opposite side of the electrochemical cell, a current response at *E* = 0.0 V *vs.* SCE of *ca.* −235 μA cm^−2^ was recorded, implying that the majority of the CAT-loaded ccMNPs were removed from the assembly. In another experiment, the system was repeatedly cycled between the magnetically attached (BOD/CAT cascade) state and the magnetically detached (only BOD responsive) state. The results indicated that through the magnetically induced shuttling of the CAT-modified ccMNPs, the system could be altered reversibly and seamlessly between the two catalytic states, see Fig. S2 in the ESI† for full details. The above results, depicted in Fig. 1, indicate that: (i) an external trigger, such as a magnetic field gradient, can be effectively used to assemble, and dissociate, enzymatic assemblies on demand. (ii) A careful engineering of the system, including the interface between the two enzymatic counterpart elements, results in direct charge transfer and bioelectrocatalysis. The magnet-induced association of the cascadic counterparts increases the levels of the enzymatically generated O_2_ (substrate 1) in the vicinity of the primary layer, due to shortening of the diffusion pathways for the generating catalyst enzyme 2. The relatively high current responses evident are also attributed to the dual layer assembly that avoids competition between the corresponding enzymes over the coverage of a single electrode surface, as well as the high intrinsic electronic conductivity of the mpCNPs and their good adhesion to the GC collector. (iii) The resulting assembly is operated by different substrates whose combined enzymatic transformations lead to intensified bioelectrocatalysis, promising for applications aimed at harvesting energy from fuel mixtures. (iv) As a side benefit related to the design, the cascadic assembly operates under changing environmental conditions, *e.g.* under both aerobic and anaerobic conditions, where the latter are introduced with H_2_O_2_ dissolved in a N_2_-saturated buffer. Along with these observations, we would like to note that no effect of the magnetic field on the bioelectrocatalysis was evident in our case, as experimentally verified and discussed in Fig. S3 of the ESI.† Also, to complement our experiments, we have tested a configuration based on an entire magnetic attachment of both BOD and CAT. This was carried out by replacing the physical adsorption of the primary BOD-mpCNPs with the magnetic immobilization of BOD-modified ccMNPs onto the GC collector. Whereas the loading of BOD was found to be *ca.* 18% lower on the magnetic layer as compared to the primary layer, similar normalized responses were obtained in the presence of O_2_ or O_2_ and H_2_O_2_ for both configurations, Fig. S3, curves (a) and (c), ESI.† Although these results demonstrate the versatility in the design and modularity of the cascadic assembly, one should remember that the all-magnetized configuration cannot be reversibly detached and reactivated upon the application of the magnetic source, due to the loss of the internal ordering of the primary and secondary layers, as demonstrated in Fig. S3, curve (d).†

The successful implementation of the magnetically induced enzymatic cascade for the generation of multi-substrate driven bioelectrocatalysis was further exemplified in two anodic systems. Fig. 1(B) depicts the voltammetry responses obtained, under different conditions, for an assembly composed of a base layer of mpCNPs loaded with fructose dehydrogenase (FDH). This enzyme has been previously shown to support DET upon adsorption to carbonaceous matrices,^27,29,30^ which follows the wiring of its flavin adenine dinucleotide (FAD) cofactor to the conductive carbon support. The chemical reaction catalyzed by FDH is given by:

3 β-d-Fructose → 5-dehydro-d-fructose + 2e^−^ (on Enz_1_ = FDH)

The second part of the assembly consists of ccMNPs modified with invertase (INV), which catalyzes the hydrolysis of sucrose, as shown in eqn (4), and formation of the β-d-fructose product, serving as a substrate for the FDH bioelectrocatalyst.

4 Sucrose + H_2_O → β-d-fructose + α-d-glucose (on Enz_2_ = INV)

In a set of experiments analogous to those performed for the BOD/CAT system, it is shown that upon the magnetically directed association of the INV-functionalized ccMNPs to the FDH-loaded mpCNPs and the introduction of either of the substrates fructose, curve (Enz_12_S_1_), or sucrose, curve (Enz_12_S_2_), bioelectrocatalytic currents with an onset potential of *ca.* −0.15 V *vs.* SCE, are formed. A similar onset value was previously described for FAD-based FDH immobilized on carbonaceous surfaces.^31–33^ The anodic currents, which intensify with the respective sugar concentration in the system, in accordance with eqn (3), correspond to the DET oxidation of fructose, either directly introduced or enzymatically produced in the vicinity of the FDH base layer. As the redox potential of the FAD cofactor of FDH corresponds to *E*^o^ = −0.48 V *vs.* SCE, the resulting bioelectrocatalytic current is obtained at an overpotential of *ca.* 330 mV. Following equilibration, the comparison between the current responses tested for the two sugars on the FDH/INV cascadic assembly yielded close results, 580 μA cm^−2^ for fructose *vs.* 500 μA cm^−2^ for sucrose, both 200 mM, in agreement with the 1 : 1 stoichiometry between the sugars in the enzymatic transformation described in eqn (4). The slightly lower current generated by sucrose is explained by inevitable losses of the fructose formed on the INV layer through diffusion to the solution. Furthermore, one can also notice that the catalytic response of the magnetically induced cascade in the presence of fructose, curve (Enz_12_S_1_), is slightly lower in comparison to testing this substrate on the FDH-modified primary layer alone, Fig. 1(B) inset, curve (Enz_1_S_1_). This observation is attributed to a minute blocking effect of the magnetic nanoparticles, which, upon magnetization to the primary layer, limit the diffusion pathways of fructose to the FDH biocatalyst. Whereas the activation of the electrocatalysis using sucrose alone, curve (Enz_12_S_2_), provides by itself direct evidence for the formation of a fully functioning magnetically assembled FDH/INV cascadic assembly, the addition of both the substrates fructose and sucrose, both 200 mM, to the magnetized cascade construction, curve (Enz_12_S_12_), resulted in an elevated bioelectrocatalytic response that reached 780 μA cm^−2^. It should be noted that this response is lower than the sum of current contributions by the two separate substrates on the cascadic assembly. As the local concentration of fructose near the primary layer is increased by the enzymatic transformation at the INV-functionalized ccMNPs in the fructose-containing electrolyte, FDH is saturated by its own substrate, which leads to the current limitation observed. To emphasize the generic nature of the presented methodology, a third example showing the magnetically assisted induction of cascaded bioelectrocatalysis is demonstrated. In this example we aimed to show the applicability of the concept to mediated electron transfer, MET, assemblies. To this end, we used an enzymatic cascade composed of a primary layer modified with horseradish peroxidase, HRP. HRP typically requires a donor to facilitate its hydrogen peroxide reduction transformation. In our case, we exploited the properties of the mpCNP matrix, allowing the entrapment of the oxidized donor molecules in the form of methylene blue, MB^+^, inside the nanopores.^20^ Following the capping of the matrix with GOx, a stable configuration is obtained in which MB acts as a mediator for the enzymatic reaction through the regeneration of the cofactor. This is achieved through the electrochemical reduction of MB^+^, which facilitates a subsequent enzymatic reaction, as depicted in eqn (5a) and (5b), respectively:

5a MB^+^ + e^−^ → MB

5b H_2_O_2_ + MB → H_2_O + MB^+^ (on Enz_1_ = HRP)

Following the reduction of the enzymatically generated MB^+^ near the electrode surface, a bioelectrocatalytic current is formed in the system. In order to trigger a magnetically induced cascade using this assembly, we have conjugated it to a counterpart enzyme, glucose oxidase, GOx, which oxidizes glucose simultaneously with reducing its native acceptor O_2_ to the HRP's substrate H_2_O_2_.

6 β-d-Glucose + O_2_ → d-glucono-1,5-lactone + H_2_O_2_ (on Enz_2_ = GOx)

Upon the magnetization of the GOx-functionalized ccMNPs to the HRP/MB^+^-modified mpCNPs on the surface, a catalytically effective cascade structure was obtained, Fig. 1(C). Interestingly, the recorded bioelectrocatalytic currents do not reach the typical steady state characteristics expected for a mediated electron transfer system. We have revealed that the reason for this observation lies in an accompanying process of direct reduction of H_2_O_2_ on the mpCNP matrix itself, Fig. S4.† Also evident in Fig. 1(C) is that the onset potential of electrocatalysis is significantly more positive than the redox potential of the pore-entrapped methylene blue mediator. While this phenomenon is not fully clear, we have observed that upon activation of the same cascade system, yet by introducing MB as the soluble electrolyte species, a good overlapping between the catalytic reduction onset and the mediator potential was observed, Fig. S5.† Notably, also no DET in the absence of MB was observed in our system. We thus suspect that the confinement of the oxidized mediator in the nanopores has an effect on its acceptor characteristics in the catalytic process. Furthermore, we have verified that methylene blue does not support glucose oxidation through mediation of the magnetized, GOx-functionalized ccMNPS, Fig. S6,† and that no electropolymerization of this species^34–37^ is possible within the scan range shown for catalysis.

At this stage, we have decided to further demonstrate the applicability of the system in the construction of a magnetically assembled cascadic biofuel cell. The cell combined the anodic FDH/INV and cathodic BOD/CAT constructions to yield a multiple fuel/oxidizer-driven, all-DET biofuel cell. Fig. 2(A) presents the discharge polarization curves obtained upon the discharge of the FDH/INV//BOD/CAT cell against variable external resistances, upon magnetization of the respectively modified ccMNPs, and in the presence of either fructose and O_2_, curve (a), or their mixture with sucrose and H_2_O_2_, curve (b). Evidently, in both cases the open circuit voltage reached *ca.* 0.62 V, in agreement with the difference in the onset potentials for electrocatalysis demonstrated in Fig. 1(A) and (B) for these systems. Whereas the introduction of substrates solely activating the bioelectrocatalysis associated with the primary layers resulted in a steep discharge profile, the addition of the secondary layers' fuel and oxidizer to the electrolyte induced the cascadic functions, which decreased the polarization and supported an elevated cell performance. Furthermore, the discharge characteristics of the biofuel cells follow the electrocatalytic responses by the respective half-cell reactions. As compared to the operation of the cell using the base stationary layer only, the magnetization of the cascade layer and the addition of the secondary layers' fuel and oxidizer to the electrolyte lead to a decrease in the observed cell polarization losses with a concurrent increase in the power output, as evident in Fig. 2. These reflect the increase in bioelectrocatalytic currents in Fig. 1. Under these conditions, the FDH/INV anode is the charge-limiting electrode in the cascadic biofuel cell as it demonstrates a 552 μA cm^−2^ V^−1^ enhancement yielding a polarization value of 1.31 mA cm^−2^ V^−1^ that is 21% lower than the polarization observed for the BOD/CAT cathode. Translation of the discharge characteristics into power outputs reveals an increase of *ca.* 138% in the maximal power density between the two cases, curves (a) and (b), with a power density of *P* = 166 μW cm^−2^ for the discharge of the fully activated cascadic cell, Fig. 2(B). This performance exceeds that of most reported sugar or alcohol fueled enzymatic biofuel cells,^15,38–44^ yet leaves room for improvement compared to other fuel oxidations *via* enzymatic cascades,^45,46^ some extracting up to 24 e^−^ per substrate molecule.^47–50^ When putting our results into perspective one should take into account that (i) unlike almost all other studies, in our biofuel cell both electrodes were operated through enzymatic cascades; (ii) whereas the direct loading of the enzymes is in many cases not reported, in our study it was much lower than on, *e.g.* carbon felt or nanotube modified electrodes;^42–44^ (iii) no mediator molecules were incorporated into our assembly in contrast to other similar constructs;^19,44,51,52^ and (iv) we applied no mechanical stirring or rotation to the fuel cell. Furthermore, our approach using a cathodic cascade enables the use of H_2_O_2_ as an internal oxidizer source, which significantly enhances the power outputs and paves the way for future potent enzymatic biofuel cells.

**Fig. 2:**
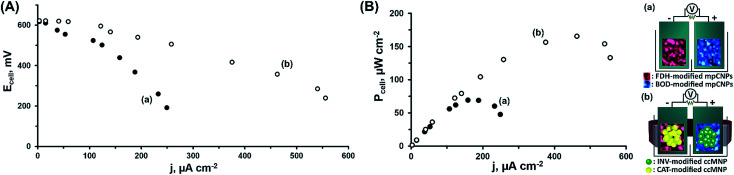
(A) The discharge polarization curves measured for the all-DET biofuel cell operating on: (a) 200 mM fructose and saturated O_2_ on the primary layer of FDH-modified mpCNPs on the anode and BOD on the cathode, and (b) 200 mM sucrose and 200 mM fructose as fuel sources, and saturated O_2_ plus 40 mM H_2_O_2_ as oxidizer sources with the additional secondary layer of INV (anodic) and CAT (cathodic) functionalized ccMNPs, activated by the application of an external magnetic field. (B) Power outputs of the cell under the conditions described in (A).

## Conclusions

We have demonstrated the use of enzyme-loaded ccMNPs for the activation of cascaded bioelectrocatalysis and biofuel cells. The on-demand magnetically induced shuttling of various enzyme-modified particles towards electrode surfaces functionalized with their respective cascade counterparts facilitated an electrical communication between the two magnetically conjugated enzymatic systems. Upon the introduction of the respective substrates into the cell, transfer of the redox electrons involved in the enzymatic transformations to/from the electrode surface was achieved, leading to a favorable energetic downhill-cascaded bioelectrocatalysis, whose intensity relates to the substrate composition in the cell. We have demonstrated that this methodology (i) is generic and can be used to activate both mediated and direct electron transfer of anodic or cathodic directionality, (ii) is efficient in broadening the spectrum of substrates contributing to bioelectrocatalysis, (iii) can be used, upon a dissociative magnetic stimulus, to instantly restore a classical, single-substrate operation for each assembly, (iv) when tuned to the specific components, shows versatility in operating under various external environments, *e.g.* aerobic or anaerobic in the case of the BOD/CAT cascade, and (v) is effective in the generation of electrical power, as exemplified by the FDH/INV//BOD/CAT magnetically cascaded biofuel cell delivering 166 μW cm^−2^. Through the careful and logical selection of the elements involved, implementation of the assemblies can be further extended to higher order enzymatic cascades, highlighting a promising path for harvesting “clean” power from mixed biomass. Further potential uses of the presented method include, for example, its application in conjunction with electrodes carrying several co-immobilized enzymes. Through magnetic binding of the respective specific enzymatic counterparts, selective power harvesting can be achieved and tuned to the biomass content of the environment of interest. Furthermore, the triggered assembly and disassembly of biologically driven systems using an external magnetic trigger to control their functionality can be applicable to biological systems other than enzymes, with possible implications beyond bioelectrocatalysis.

## Conflicts of interest

There are no conflicts to declare.

## Supplementary Material

NA-001-C8NA00346G-s001
