# Supplementary material for: Magnetically induced enzymatic cascades – advancing towards multi-fuel direct/mediated bioelectrocatalysis
Source: Nanoscale Adv. 2019 Feb 28;1(5):1686–92. doi: 10.1039/c8na00346g (PMC9419066; doi:10.1039/c8na00346g)
Supplement: NA-001-C8NA00346G-s001 [file NA-001-C8NA00346G-s001.pdf]

Electronic Supplementary Material (ESI) for Nanoscale Advances.
This journal is © The Royal Society of Chemistry 2019

Electronic Supplementary Information

Magnetically induced enzymatic cascades – advancing towards multi-fuel direct/mediated bioelectrocatalysis

Katharina Herkendell, Andreas Stemmer and Ran Tel-Vered*

ETH Zürich, Nanotechnology Group, Säumerstrasse 4, CH-8803 Rüschlikon, Switzerland.

E-mail: teran@ethz.ch

Synthesis of the active material

Primary layer

Glassy carbon (GC) electrodes (15x8x1 mm; Sigma) were polished with 1.0 µm alumina powder on a 1200 grit carbimet disk, followed by their successive polishing with 0.3 and 0.05 µm alumina particles on a microcloth pad and rinsing with ethanol and water. A 10 mg ml^{-1} mesoporous carbon particles (mpCNP, Sigma, d<500 nm, 64 Å average pore diameter, ca. 193 m^2 g^{-1}) suspension was prepared by mixing the particles for 5 minutes with N-methyl-2-pyrrolidinone (NMP), followed by 120 minutes sonication. The stock mixture of assembly III was prepared by adding the redox relay methylene blue (MB, Sigma) to the base suspension to yield a final concentration of 2 mM mediator molecules per suspension volume. Following sonication for 120 minutes the stock solutions were introduced with 50 µl of poly(vinylidene fluoride) (PVDF, Sigma, M_W ~ 180'000 g $mole^{-1}$) solution (5 wt% in NMP), and were further sonicated for 20 minutes. In the next stage, 8 µl of the respective mpCNP stock suspensions were applied on top of the GC surfaces, and the electrodes were left to dry for 30 minutes at 10 mbar. Subsequently, 10 µl MES buffer solution (0.1 M, pH 5.5) containing either bilirubin oxidase, BOD, from *Myrothecium*

verrucaria (Sigma), 2 mg ml^{-1}, D-fructose dehydrogenase, FDH, from *Gluconobacter sp.* (Toyobo Enzymes), 8 mg ml^{-1}, or peroxidase from *horseradish*, HRP, Sigma, 2 mg mL^{-1}, was applied on top of the mpCNP matrix. After 10 minutes the assemblies were covered by 1 µl of the cross-linking agent suberic acid bis(3-sulfo-N-hydroxysuccinimide ester) sodium salt, (BS^3, Sigma, 1 mg ml^{-1} aq.). The modified electrodes were allowed to dry at room temperature and rinsed with the buffer to remove any excess of non-bound components. The electrodes were then coated with 3 µl of Nafion™ solution (Sigma, 0.5 v% aq.) and dried at room temperature. Crosslinking and covering the primary layer assist the prevention of enzymatic disintegration due to desorption, and strengthen the mechanical stability of the modifying layer.

Secondary layer

1-pyrenemethylamine hydrochloride (Sigma, 10 mg) was dispersed in 5 mL water and sonicated for 10 minutes. To this solution, 5 mg of carbon coated magnetic nanoparticles (ccMNPs, d=25 nm, Sigma) were added, and the resulting mixture was shaken for 30 minutes. Typical scanning electron microscopy images of the nanoparticles are provided in Figure S7. The pyrenemethylamine-modified ccMNPs were washed with water and subsequently suspended in 200 µL MES buffer. In the following step 50 µL MES buffer containing either catalase, CAT, from *bovine liver* (Sigma), 1 mg ml^{-1}, invertase, INV, from *Saccharomyces cerevisiae* (Sigma), 3 mg ml^{-1}, or glucose oxidase, GOx, from *Aspergillus niger* (Sigma), 1 mg ml^{-1}, was added, and the suspension was left for 14 hours allowing the enzyme to adsorb on the surface of the modified nanoparticles through hydrophilic interactions and π-π-stacking between the carbon surface and protein regions enriched with aromatic amino acid residues. The enzyme-modified ccMNPs were magnetically collected and washed three times with the MES buffer to remove all non-bound enzymes. Control experiments employing BOD-modified ccMNPs were similarly prepared, yet discarding the pyrenemethylamine pretreatment. To induce the enzymatic cascades, 20 µL suspension containing 25 mg mL^{-1} enzyme-modified ccMNPs was magnetically attached on the top of the primary layer-modified electrode.

Electrochemical setup

Electrochemical measurements were performed using a PalmSens3 potentiostat (PalmSens BV) connected to a three-electrode setup. A KCl-saturated calomel electrode (SCE) and a carbon rod served as the reference and counter electrodes, respectively. Unless indicated, all experiments were performed in MES buffer (0.1 M, pH=5.5) at room temperature. In none of the experiments mechanical stirring was applied. The enzymatic biofuel cells were discharged versus variable external resistances using a Keithley 2000 multimeter. The modified enzyme 1-mpCNP-GC primary layer electrodes were mounted inside a 5 mL cell, for both, the half-cell and the full fuel cell testings. The footprint of the assemblies on the current collectors was 0.32 cm^2, which was equal to the surface area of the GC current collectors immersed in the electrolyte.

Spectroscopic assays

UV/Visible spectroscopic assays were performed on a Bio-Rad SmartSpec Plus spectrophotometer using quartz cuvettes and employing 2-(N-morpholino)ethanesulfonic acid (MES) buffer (0.1 M, pH = 5.5). For each enzymatic assay, several concentrations of the enzymes were first separately analyzed. Following the measurement of the absorbance of the respective marker molecule in the cuvettes after an equilibration time of 5 minutes, linear calibration curves were obtained for the enzymes, correlating the absorbance to the mass of the active enzymes. Upon testing the enzyme-modified electrode surface under conditions similar to the assay, an absorption value was derived, and correlated to the calibration curve, allowing the estimation of the surface coverage of the enzyme on the electrode, as described in Table S1.

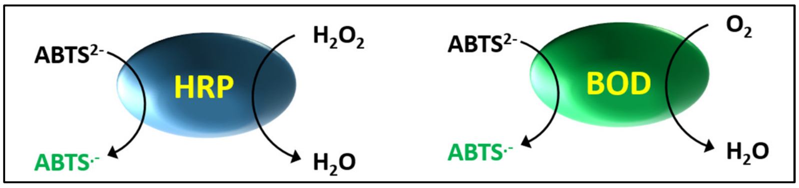

Fig. S1 (A) *Mechanism of assaying the amount of active HRP and BOD.*

The loading of the surface-bound BOD and HRP was quantified by following the BOD-catalyzed reduction of O_2 (saturated) to H_2O, or the HRP-catalyzed reduction of H_2O_2 (50mM) respectively, facilitating the oxidation of the colorless

2,2′-azino-bis(3-ethyl benzothiazoline-6-sulfonic acid) diammonium salt, $ABTS^{2-}$ (20 mM, Sigma) to the green absorbing $ABTS^{\cdot-}$ species (marker molecule) at λ_{max} = 414 nm, Figure S1(A).[1,2]

The amount of the catalytically active FDH immobilized on the electrodes was determined by the reduction of the yellow colored potassium ferricyanide (20 mM, marker molecule, Sigma), λ_{max} = 420 nm, to the colorless potassium ferrocyanide, serving as an electron acceptor in the FDH-catalyzed oxidation of β-D-fructose (200 mM) to 5-dehydro-D-fructose and thus supporting a decrease in the total absorbance, Figure S1(B).[3,4]

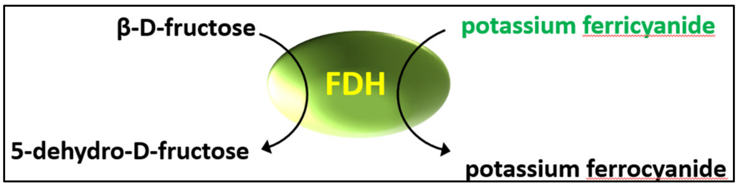

Fig. S1 (B) *Mechanism of assaying the amount of active FDH.*

The surface coverage of INV was similarly obtained by following the INV-catalyzed transformation of sucrose (200 mM) to β-D-fructose, which was, as described above, further oxidized by FDH (1 nM), with the concurrent reduction of the potassium ferricyanide marker, Figure S1(C).

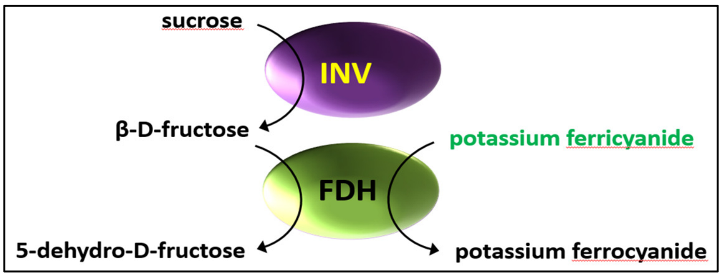

Fig. S1 (C) *Mechanism of assaying the amount of active INV.*

The amount of the catalytically active GOx immobilized on the ccMNPs was determined by following the O_2(sat.)-mediated oxidation of glucose (200 mM) to glucono-1,5-lactone. To this end, the colorimetric analysis of the enzymatically generated H_2O_2 was assayed by following the HRP (2 nM) catalyzed oxidation of $ABTS^{2-}$ (20 mM), Figure S1(D).

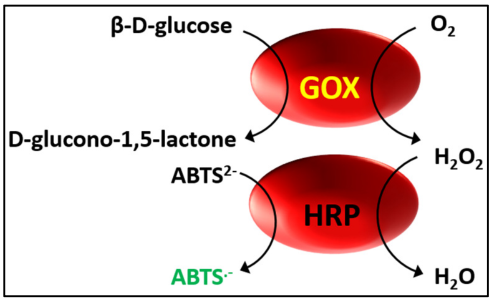

Fig. S1 (D) *Mechanism of assaying the amount of active GOx.*

Different concentrations of CAT were spectroscopically assayed in buffer solutions containing fixed levels of H_2O_2, 40 mM, HRP, 2 nM, and $ABTS^{2-}$, 20 mM. The CAT acted as an inhibitor for the HRP-catalyzed oxidation of the $ABTS^{2-}$ to the marker molecule $ABTS^{\cdot -}$, through the catalytic disproportionation of the HRP's H_2O_2 substrate to water and oxygen, Figure S1(E).

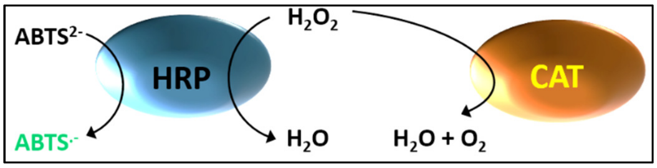

Fig. S1 (E) *Mechanism of assaying the amount of active CAT.*

Table S1 *Enzymatic loading on the electrode surfaces derived from the assays.*

**enzyme**	**MW** *kDa*	m_{enzyme}[a] *pg*	Γ_{enzyme}[b] *pmole cm*$^{-2}$	**enzyme per mpCNP support**[c] *wt%*	**enzyme per ccMNPs**[d] *wt%*
BOD	60	115	6.0±1.0	0.14	-
FDH	140	170	3.8±0.9	0.21	-
HRP	440	130	9.2±1.3	0.16	-
CAT	323	294	4.0±0.2	-	0.06
INV	135	274	6.3±1.0	-	0.06
GOx	160	176	3.4±0.4	-	0.04

a) Mass of enzyme per electrode derived from the assays described

b) Surface area A=0.32 cm^2

c) 0.5 mg ccMNPs per electrode as the secondary layer

d) 0.08 mg ccMNPs per electrode as the primary layer

Magnetic attachment/detachment experiments

We have further explored the possibility to magnetically shuttle enzymatic cascade components to and away from their surface-immobilized counterparts. Figure S2(A) depicts the bioelectrocatalytic responses by the model BOD/CAT system. Upon attracting the CAT-functionalized ccMNPs to the BOD-loaded surface the catalytic cascade was activated, which led

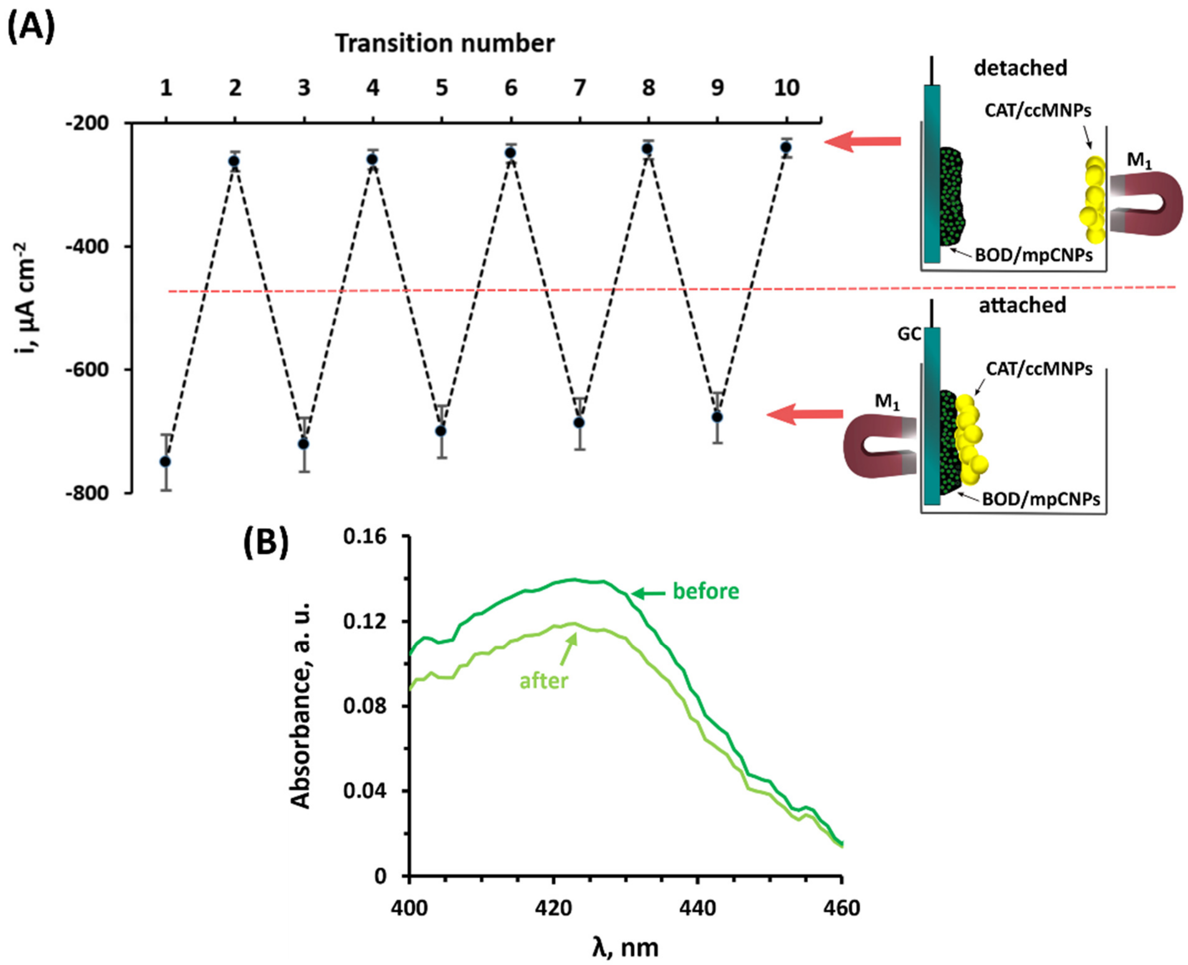

Fig. S2 *(A) Bioelectrocatalytic current densities at E=0.0 V vs. SCE corresponding to five magnetic attachment/detachment cycles of CAT-functionalized ccMNPs on BOD/mpCNPs-modified GC. Measurements were performed using cyclic voltammetry in* O_2*-saturated MES buffer (0.1M, pH 5.5) in the presence of 40 mM* H_2O_2*, with 10 minutes equilibration time of each state and a scan rate of 10 mV* s^{-1}*. (B) Spectroscopical assays of the BOD/mpCNPs-modified GC before and after the measurements in (A).*

to the generation of high current density outputs. Reversing the magnet to the other side of the cell resulted in the removal of the CAT from the surface and to a decrease in the local concentration of O_2 near the electrode (detached state), consequently leading to lower electrocatalytic currents. As can be seen, repetitive cyclic transitions between the attached and detached states yielded in both cases a 10-12% current loss over the entire experiment duration (ca. 0.1 % min^{-1}). The origin of this decrease is explained by the cumulative loss of enzyme activity during the 100 minutes measurement. To support this, we have included in Figure S2(B) the, $ABTS^{\cdot -}$ UV/Vis absorption spectra corresponding to the assay of the surface immobilized BOD

prior and following the 5 attachment/detachment cycles in Figure S2(A). The enzymatic assay, elaborated here in the ESI, indicates an enzymatic activity loss of ca. 14%, which is in a fair agreement with the reduction in the electrocatalytic responses observed. It should also be noted that due to the short shuttling distances imposed by the cell geometry, no ccMNPs were found to precipitate during the magnetic transitions thus becoming inaccessible for catalysis.

Monitoring the possible influence of the magnetic field on the bioelectrocatalysis

The effect of the magnetic field on the bioelectrocatalysis was probed by conducting the four comparative experiments depicted in Figure S3.

In all experiments, bioelectrocatalytic currents were measured and compared in the presence of either N_2, O_2, or O_2 + H_2O_2 for both magnetized and non-magnetized BOD/CAT cascade assemblies. The results indicate that the magnetic effect in our system accounts for only less than 2%, as indicated by the invariance of the catalytic currents in the presence, bars (a) and (c), or the absence of the magnet, bars (b). In the latter configuration, we used a Nafion membrane to immobilize the CAT-functionalized ccMNPs on the surface, and all current outputs were spectroscopically normalized to the loading of the BOD on the electrode surfaces. These results are in agreement with studies reporting no effect of magnetic field on enzymatic catalysis.[5,6] Also indicated in this figure is the effect of a magnetic introduction of a mixed, non-layered, BOD/CAT cascade assembly, bars (d). The decreased order led to a partial blocking of the H_2O_2 diffusion pathways towards the CAT-coated particles and lowered the resulting currents in the presence of this substrate.

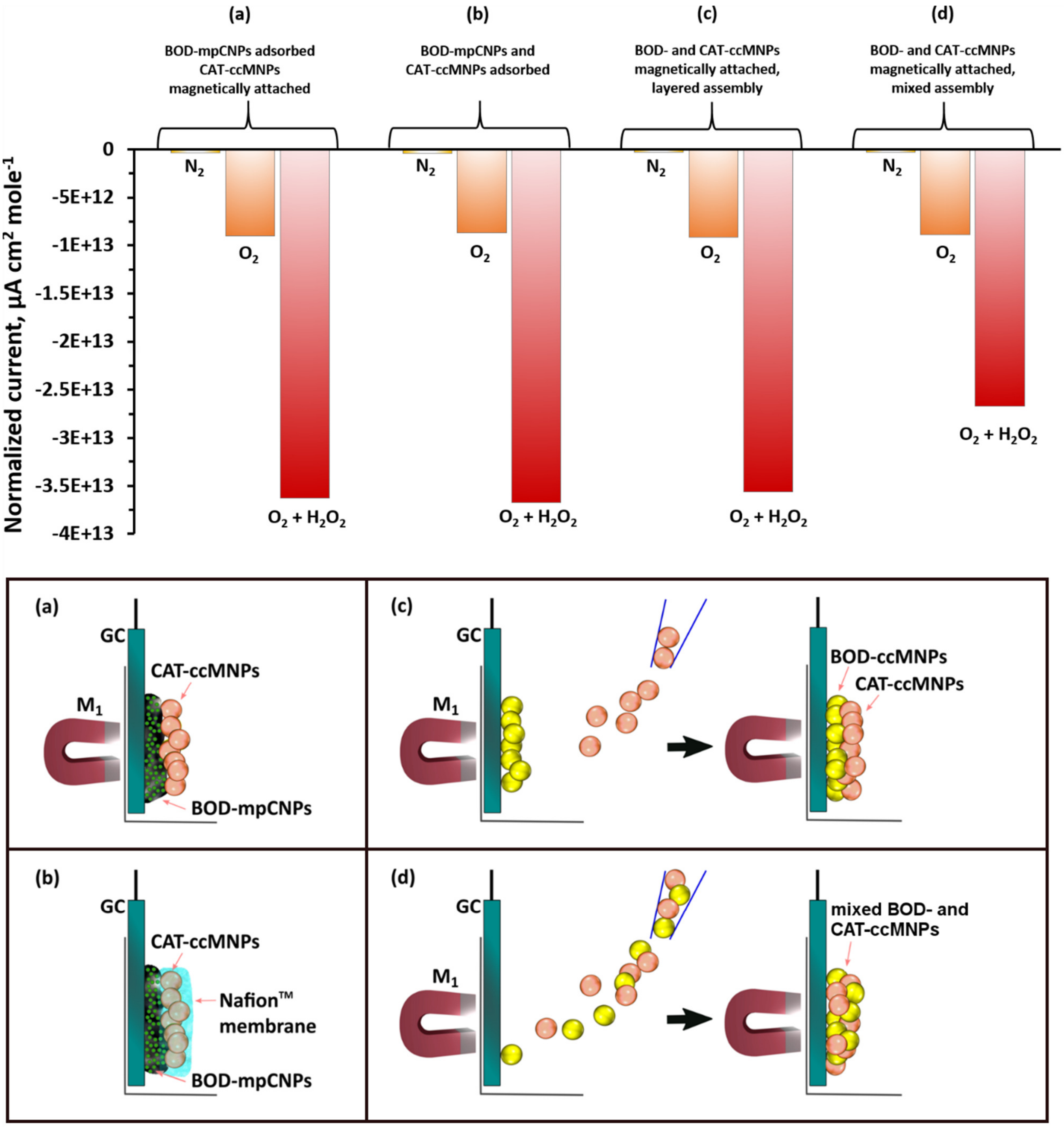

Fig. S3 *Bioelectrocatalytic responses at E=0.0 V vs. SCE, normalized by the surface coverage of active BOD, recorded for: (a) Magnetically attached CAT-modified ccMNPs on BOD-capped mpCNPs; (b) CAT-modified ccMNPs adsorbed on BOD-capped mpCNPs, stabilized by a Nafion™ layer; (c) magnetically attached CAT-modified ccMNPs on a primary layer consisting of magnetically immobilized BOD-modified ccMNPs; and (d) magnetically attached BOD- and CAT-modified ccMNPs in a mixed assembly. The different substrates employed in the measurements are indicated in the graph. Capacitance currents were subtracted for normalization purposes. Measurements were performed using cyclic voltammetry in MES buffer (0.1 M, pH 5.5), at a scan rate of 10 mV s^{-1}.*

Direct reduction of H_2O_2 on the carbonaceous matrix

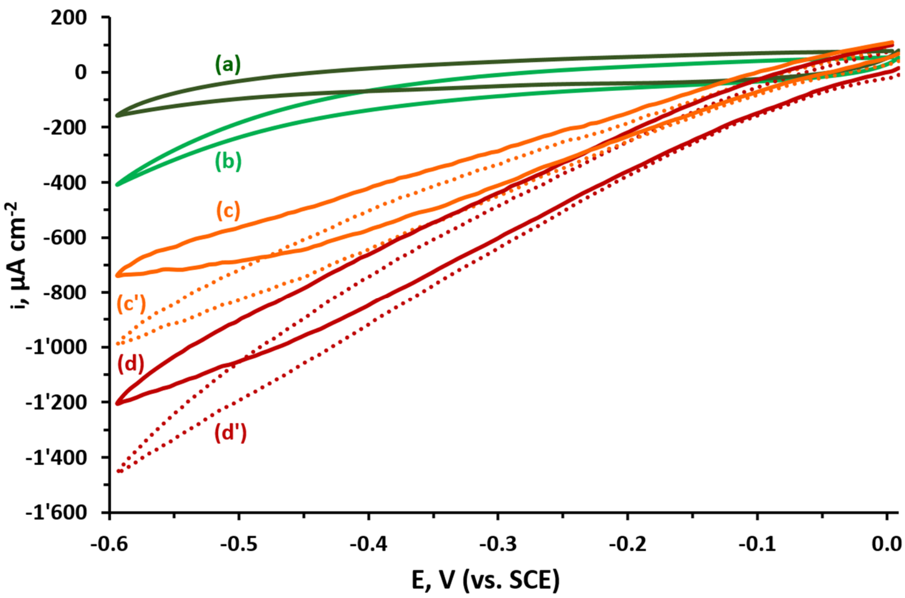

Fig. S4 *Cyclic voltammograms corresponding to: (a) mpCNPs-modified GC magnetized with enzyme-free ccMNPs in N_2-purged buffer, and (b) the voltammetric response following the addition of H_2O_2, 40 mM, to (a). The reduction current that corresponds to the difference between (b) and (a) was subtracted from curves (c') and (d') - representing the challenging of the HRP/GOx cascade system in Figure 1(C) with $E_{12}S_1$ and $E_{12}S_{12}$, to yield the corrected bioelectrocatalytic values, demonstrated in curves (c) and (d), respectively.*

Bioelectrocatalytic currents associated with the reduction of H_2O_2 in freely diffusing solubilized methylene blue

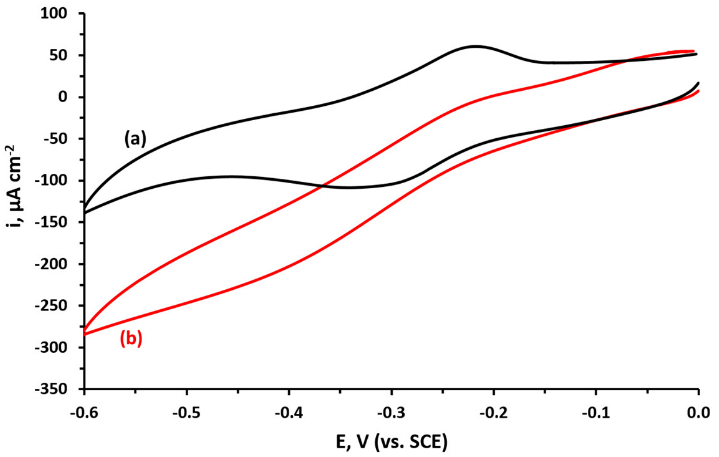

Fig. S5 *Cyclic voltammograms corresponding to the magnetically attached HRP/GOx cascade system on a mediatorless mpCNPs matrix in the presence of electrolyte solubilized MB in the: (a) absence, and (b) presence of glucose, 200 mM. The measurements were performed at a scan rate of 10 mVs^{-1} in an O_2-saturated MES buffer.*

Incapability of methylene blue to serve as a mediator for the GOx catalyzed oxidation of glucose

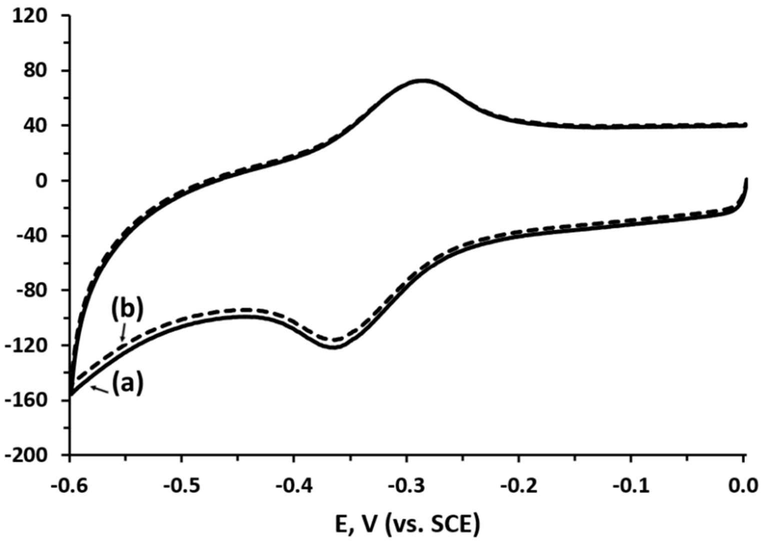

Fig. S6 *Cyclic voltammograms corresponding to the magnetically attached HRP/GOx cascade system on the pore-entrapped MB mpCNPs matrix in the: (a) absence, and (b) presence of glucose, 200 mM. The measurements were performed at a scan rate of 10 mV*s^{-1} *in a* N_2*-saturated MES buffer.*

Scanning electron microscopy images on the ccMNPs

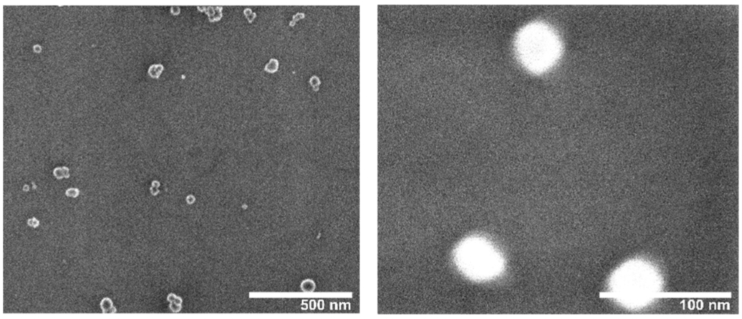

Fig. S7 *Scanning electron microscopy images corresponding to ccMNPs adsorbed on a Si wafer.*

References (ESI)

1 A. Trifonov, K. Herkendell, R. Tel-Vered, O. Yehezkeli, M. Woerner and I. Willner, *ACS Nano*, 2013, **7**, 11358.

2 K. Otsuka, T. Sugihara, Y. Tsujino, T. Osakai and E. Tamiya, *Anal. Biochem.*, 2007, **370**, 98.

3 M. Ameyama, E. Shinagawa, K. Matsushita and O. Adachi, *J. Bacteriol.*, 1981, **145**, 814.

4 K. Herkendell, R. Tel-Vered and A. Stemmer, *Nanoscale*, 2017, **9**, 14118.

5 H. L. Messiha, T. Wongnate, P. Chaiyen, A. R. Jones and N. S. Scrutton, *J. R. Soc., Interface*, 2015, **12**, 20141155.

6 K. Herkendell, A. Stemmer and R. Tel-Vered, *Nano Res.*, 2019, DOI: 10.1007/s12274-019-2285-z.
